# Bearing Performance Degradation Assessment Based on SC-RMI and Student’s t-HMM

**DOI:** 10.3390/ma14206077

**Published:** 2021-10-14

**Authors:** Huiming Jiang, Jinhai Luo, Bohua Zhou, Chao Li, Zhongwei Lv, Zhibo Yang, Jin Chen

**Affiliations:** 1 School of Mechanical Engineering, University of Shanghai for Science and Technology, Shanghai 200093, China; luo_jinhai@163.com; 2 Shanghai Academy of Spaceflight Technology, Shanghai 201109, China; usst2021@163.com; 3 Shanghai Radio Equipment Institute, Shanghai 200438, China; usst2023@163.com (C.L.); usst2022@163.com (Z.L.); 4 School of Mechanical Engineering, Xi’an Jiaotong University, Xi’an 710049, China; 5 School of Mechanical Engineering, Shanghai Jiao Tong University, Shanghai 200240, China; jinchen@sjtu.edu.cn

**Keywords:** performance degradation assessment, feature selection, spectral clustering, rank mutual information, Student’s t-HMM

## Abstract

Bearing performance degradation assessment (PDA), as an important part of prognostics and health management (PHM), is significant to prevent major accidents and economic losses in industry. For the data-driven PDA, the extraction and selection of features is quite important. To better integrate the degradation information, the bearing performance degradation assessment based on SC-RMI and Student’s t-HMM is proposed in this article. Firstly, spectral clustering was used as a preprocessing step to cluster features with similar degradation curves. Then, rank mutual information, which is more suitable for trendability estimation of long time series, was utilized to select the optimal feature from each cluster. The feature selection method based on these two steps is called SC-RMI for short. With the selected features, Student’s t-HMM, which is more robust to outliers, was utilized for performance degradation modeling and assessment. The verifications based on an accelerated life test and the public XJTU-SY dataset showed the superiority of the proposed method.

## 1. Introduction

Rolling element bearings are widely used in rotary machines, such as drills, electric motors, wind turbines, and turbofan engines. Therefore, bearing failure may lead to abrupt shut-downs, costly losses, and even catastrophic accidents [1]. Recently, the precision and complexity of modern machinery and equipment are constantly improving. While the prognostics and health management are facing huge challenges, fault diagnosis and performance degradation assessment (PDA) are receiving more and more attention [2,3]. PDA focus on the changes in the equipment health status during the entire service process, rather than just discovering whether the equipment malfunctions. Effective PDA results are the basis for further accurate remaining useful life (RUL) prediction, which can largely ensure the safety and reliability of the equipment in the process of operation and reduce the cost of equipment maintenance [4,5]. Therefore, the research related to bearing PDA has been widely concerned [6,7,8].

Feature extraction and selection, as an important procedure in bearing PDA, directly affects the final PDA results. Many common features have been applied, including time-domain features such as root mean square (RMS) and kurtosis, frequency-domain features such as frequency kurtosis, and time-frequency domain features obtained by wavelet packet decomposition, empirical mode decomposition, and variational mode decomposition as well as other features such as those based on mathematical morphology particles [9]. Meanwhile, with the development of deep learning, many deep network architectures are introduced for feature extraction and health indicator construction, such as long short-term memory [10] and convolutional neural networks [11]. In order to solve the problem that bearing vibration signals are susceptible to serious interference, He et al. [12] proposed multi-resolution singular value decomposition and a long short-term memory-network-based bearing PDA method. Xu et al. [13] used a moving window-based stacked auto-encoder with an exponential function to construct a smooth degradation curve. The deep-learning-based methods can realize end-to-end deep feature extraction without human intervention automatically. However, time-consuming and unclear physical meaning are the common shortcomings of deep learning.

**Figure 1:**
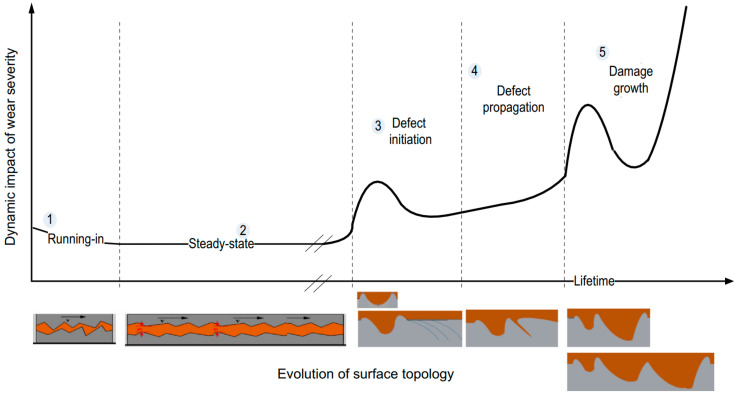
The whole lifetime wear evolution of rolling bearing.

With the rapid development of signal processing and feature extraction technique, the high-dimension features containing abundant fault and degradation information can be available. In this case, how to extract or select features with high degradation information and low redundancy becomes the key issue. Typical metrics such as monotonicity, robustness, trendability, and consistency are often used for feature evaluation. Niu et al. [14] utilized the rank mutual information criterion to measure the nonlinear correlation between feature and time. Chen et al. [15] calculated the mixed scores of three evaluation indicators and utilized a variant correlation-based feature selection method to determine the number of optimal features. Methods based on mixed evaluation indicators can more comprehensively evaluate features without doubt, but the parameters of mixed evaluation metrics commonly rely on manual experience adjustment, which limits its application. Besides, the whole lifetime of bearings contains several different degradation stages. Taking the evolution process of bearing wear fault as an example, this characteristic of bearing degradation process can be shown directly in Figure 1 provided by the literature [16]. The surface roughness becomes smoother in the running-in stage, and the steady-state stage is accompanied by uniform lubricating film and contact mechanics. In the third stage, microcracks initiate and open on and under the surface. Then, the microcracks gradually expand to produce secondary cracks or separation. The evolution mechanism of each degradation stage is different, which leads to different vibration characteristics. Therefore, the evaluation of degradation features can not only be estimated from the overall development trend but also considering the local structure of feature curves. However, in the existing literature, this factor is rarely considered in the evaluation metrics for degradation feature selection.

To solve this problem, a systematic degradation feature selection method based on spectral clustering and rank mutual information is proposed in this article. As a typical clustering algorithm, the spectral clustering algorithm can find clusters at any space and converge to the global optimal solution [17]. It is often used in fault diagnosis [18] and fault state recognition [19] but rarely in feature clustering. In this study, spectral clustering was used as a preprocessing step of feature selection to cluster features with similar degradation curves. Then, the optimal feature set was constructed by selecting the optimal feature from each cluster. Feature selection based on these two steps can not only reduce the information redundancy caused by similar feature curves in the feature set but can also ensure the diversity of different degradation curves in the feature set. In particular, how to evaluate the sensitivity of different feature curves in each cluster is the key procedure. In order to better evaluate features, rank mutual information (RMI) was introduced in this study, which is more suitable for trendability estimation of long time series. This feature selection based on these two steps is described in detail in the following sections.

After feature extraction and selection, the final PDA needs to fuse these selected multi-dimension features to build a health indicator [20]. Tse et al. [21] used principal component analysis (PCA) to construct a health indicator for impeller PDA. Guo et al. [10] input the feature set formed by integrating similar features and classical time-frequency features into a recurrent neural network (RNN) to construct RNN-HI. Different from deep learning, the hidden Markov model (HMM) infers the hidden state change through the observations, which is more suitable for PDA. HMM has been widely used in recent years due to the advantages of high accuracy in small samples and with clear physical meaning. Ocak et al. [22] used HMM for bearing fault detection and diagnosis for the first time. Yu et al. [23] proposed a machine health degradation assessment method based on HMM and contribution analysis. Li et al. [24] used the time-dependent state transition probability matrix with degradation characteristics to obtain the HMM reliability curve and realized the reliability evaluation of wind turbine components based on small sample data. Li et al. [25] established a hazard model describing the time-varying and conditional adaptive state transition probability to estimate the wear state of the tool. Yu et al. [26] proposed an adaptive-learning-based method for machine faulty detection and health degradation monitoring, which provide a useful guide for developing a condition-based maintenance system. Jiang et al. [27] combined Student’s t-HMM with nuisance attribute projection to construct a robust PDA model, which shows more tolerance to outliers than conventional HMMs. In this study, Student’s t-HMM was utilized to construct a health indicator based on the selected feature sets and to assess the degradation process.

The rest of the article is structured as follows. The related theoretical backgrounds of spectral clustering, rank mutual information, and Student’s t-HMM are introduced in Section 2. In Section 3, the whole procedures of the proposed bearing PDA are introduced. In Section 4, two experimental data sets are used to verify the proposed method. Finally, a conclusion is carried out in Section 5.

## 2. Theory Background

### 2.1. Spectral Clustering

Different from the traditional clustering algorithms like k-means, the spectral clustering algorithm is based on graph theory [28]. Spectral clustering takes samples as vertices and the similarity between samples as the weight of vertex connection edge, transforming the clustering problem into the partition problem of an undirected graph with weights. It can find clusters at any space and converge to the global optimal solution [29], which is superior to the traditional clustering method [30]. Therefore, spectral clustering is utilized to cluster different lifetime curves as a pre-procedure of feature selection in PDA.

For the sample data $\left\{x_{1},x_{2},\ldots ,x_{n}\right\}$, each data point $x_{i}$ can be represented as a vertex $v_{i}$. Let G=(V,E) be an undirected graph with a vertex set $V=\{v_{1},v_{2},\ldots ,v_{n}\}$ [22]. Assume that the graph *G* is weighted. Each edge between two vertices $v_{i}$ and $v_{j}$ carries a non-negative weight $w_{ij}$ ≥ 0. Then, a weighted adjacency matrix of the graph can be obtained as follows:

(1) $$W=\{w_{ij}\}(i,j=1,\ldots ,n)$$

As *G* is undirected, $w_{ij}=w_{ji}$. If $w_{ij}=0$, it means that the vertices $v_{i}$ and $v_{j}$ are not connected by any edge. Then, W(,) defines the relations of two not necessarily disjoint sets *A*,*B* ⊂ *V*.

(2) $$W(A,B):=\underset{i\in A,j\in B}{\sum }w_{ij}$$

The goal of spectral clustering is to cut the graph *G* = (*V*,*E*) into *k* subgraphs with no connection, which can be defined as follows:

(3) $$cut(A_{1},A_{2},\ldots ,A_{k})=\frac{1}{2}\overset{k}{\underset{i=1}{\sum }}W(A_{i},{\bar{A}}_{i})$$

The set of each subgraph points is defined as *A*_1_, *A*_2_, …, *A_k_*, and they satisfy *A_i_*∩*A_j_* = ∅, and *A*_1_∪*A*_2_∪…∪*A_k_* = V. Many techniques have been proposed to solve the cutting graph problem, and the *NCut* technique was utilized in this study [22]. Based on the *NCut* technique, the problem described in Equation (3) can be rewritten as

(4) argmin⏟Ftr(YTD−12LsymD−12Y)s.t.YTY=I

where the degree matrix D is defined as the diagonal matrix with the degrees $d_{1},d_{2},\ldots ,d_{n}$ on the diagonal, and $d_{i}=\overset{n}{\underset{j=1}{\sum }}w_{ij}$. L*_sym_* is the normalized graph Laplacian matrix defined as Lsym=I−D−12WD−12. This is the standard trace minimization problem, and solution Y consists of the minimum first *k* eigenvectors of the matrix L*_sym_*. Now, all sample points belonging to the same cluster were mapped from the original high-dimensional feature space into a new low-dimensional feature space (i.e., $x_{i}\to y_{i}$), which enabled us to obtain the final clustering result based on the simple clustering method. In this study, the classical k-means method was adopted.

### 2.2. Rank Mutual Information

The Spearman coefficient, as one of the major statistical correlation coefficients, was often used to measure the correlation between two continuous variables. Generally, the Spearman coefficient is utilized to evaluate the non-linear correlation between feature and time series for feature selection in PDA as a similarity metric. However, the whole degradation process of the bearing usually covers several different degradation stages. The evolution mechanism of each stage was different, resulting in different development patterns of vibration signals in each stage. For example, during the whole lifetime of the bearing, the normal stage is usually long and stable, while the fault stage usually changes rapidly. Unfortunately, the Spearman coefficient can only evaluate the overall trendability of time series, which cannot reflect the local structure of data. Therefore, it is not suitable as an evaluation metric for the selection of bearing degradation features. In this study, we introduced a new evaluation metric for degradation feature selection called rank mutual information (RMI). 

RMI is a generalization method based on Shannon entropy, which can be used to measure the correlation between two sequence data. In particular, when RMI is used to measure the non-linear correlation between sequence data and time series, we find that it is more easily affected by the trendability of data in the later stage. This quality has advantages in optimizing the bearing degradation feature curves. So, RMI was introduced as the evaluation metrics for feature selection in this study.

Let $X=\left\{x_{1},\cdots ,x_{n},\cdots x_{N}\right\}$ and $Y=\left\{y_{1},\cdots ,y_{n},\cdots y_{N}\right\}$ be two sequence data, where 1≤n≤N. Given $\forall x_{i}\in X$, $\forall y_{i}\in Y$, we defined the following sets:

(5) $${\left[x_{n}\right]}_{\geq }=\{x_{i}\in X|x_{i}\geq x_{n}\}$$

(6) $${\left[x_{n}\right]}_{\leq }=\{x_{i}\in X|x_{i}\leq x_{n}\}$$

(7) $${\left[y_{n}\right]}_{\geq }=\{y_{i}\in Y|y_{i}\geq y_{n}\}$$

(8) $${\left[y_{n}\right]}_{\leq }=\{y_{i}\in Y|y_{i}\leq y_{n}\}$$

Then, the ascending rank mutual information (ARMI) and the descending rank mutual information (DRMI) between sequence *X* and *Y* were defined as:

(9) $$\mathrm{ARMI}(X,Y)=-\frac{1}{N}\overset{N}{\underset{n=1}{\sum }}\log\frac{\left|{\left[x_{n}\right]}_{\leq }\right|\times \left|{\left[y_{n}\right]}_{\leq }\right|}{N\times \left|{\left[x_{n}\right]}_{\leq }\cap {\left[y_{n}\right]}_{\leq }\right|}$$

(10) $$\mathrm{DRMI}(X,Y)=-\frac{1}{N}\overset{N}{\underset{n=1}{\sum }}\log\frac{\left|{\left[x_{n}\right]}_{\geq }\right|\times \left|{\left[y_{n}\right]}_{\geq }\right|}{N\times \left|{\left[x_{n}\right]}_{\geq }\cap {\left[y_{n}\right]}_{\geq }\right|}$$

In ARMI, $\left|{\left[x_{n}\right]}_{\leq }\right|=\sum {}_{i}r_{ni}$ is the cardinality of set ${\left[x_{n}\right]}_{\leq }$; ∩ is an intersection operator; and *r_ni_* is the degree of *x_n_* worse than *x_i_*. We have:

(11) $$\{\begin{array}{c} r_{ni}=0,\;\mathrm{if}\;x_{n}>x_{i}\\ r_{ni}=1,\;\mathrm{if}\;x_{n}\leq x_{i} \end{array}$$

In DRMI, *r_ni_* is the degree of *y_n_* better than *y_i_*. We have:

(12) $$\{\begin{array}{c} r_{ni}=0,\;\mathrm{if}\;y_{n}<y_{i}\\ r_{ni}=1,\;\mathrm{if}\;y_{n}\geq y_{i} \end{array}$$

When RMI is used to measure the trendability between feature sequence *X* and time series *T*, Equations (9) and (10) could be rewritten as:

(13) $$\mathrm{ARMI}(X,T)=\frac{1}{N}\overset{N}{\underset{n=1}{\sum }}\log\frac{N\times \left|{\left[x_{n}\right]}_{\leq }\cap {\left[t_{n}\right]}_{\leq }\right|}{(N-n+1)\times \left|{\left[x_{n}\right]}_{\leq }\right|}$$

(14) $$\mathrm{DRMI}(X,T)=\frac{1}{N}\overset{N}{\underset{n=1}{\sum }}\log\frac{N\times \left|{\left[x_{n}\right]}_{\geq }\cap {\left[t_{n}\right]}_{\geq }\right|}{n\times \left|{\left[x_{n}\right]}_{\geq }\right|}$$

It can be seen from Equation (13) that the larger *n* is, the larger *N*/(*N* − *n* + 1) is. ARMI is more susceptible to the current rank sequence. From Equation (14), we can see that as *n* increases, DRMI is less affected by the current rank sequence, which is just opposite to ARMI. Therefore, we used ARMI to assess the trendability of the degradation feature sequence, aiming to select features with better trendability in the degradation or fault stage. It was easy to deduce that 1 is the upper bound of ARMI, and the derivation process is as follows:

$$\mathrm{ARMI}(X,T)\leq \frac{1}{N}\overset{N}{\underset{n=1}{\sum }}\log\frac{N\times \left|{\left[x_{n}\right]}_{\leq }\right|}{(N-n+1)\times \left|{\left[x_{n}\right]}_{\leq }\right|}=\frac{1}{N}\overset{N}{\underset{n=1}{\sum }}\log\frac{N}{\left(N-n+1\right)}\leq 1$$

**Figure 2:**
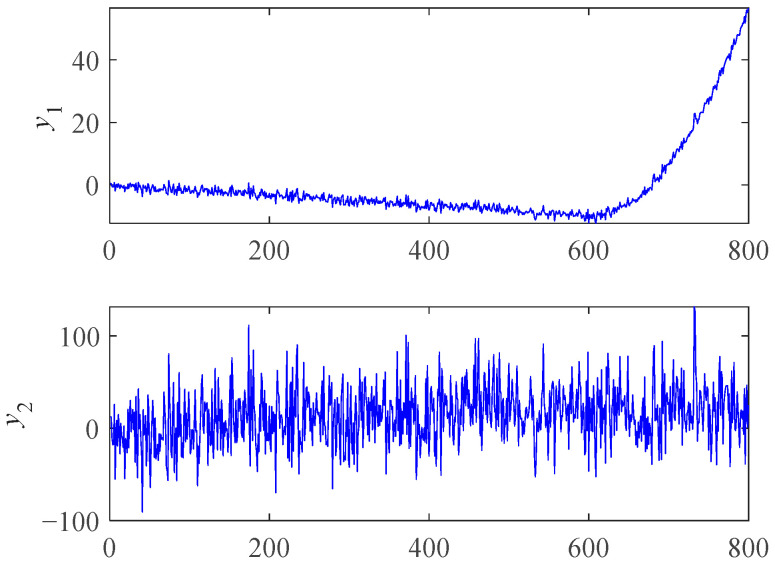
Plots of simulation data.

We used a few simple simulation data to illustrate the properties of RMI. The mathematical formulas of $y_{1}$ and $y_{2}$ are shown as follows:

(15) $$\begin{array}{l} y_{1}=\{\begin{array}{cc} -\frac{x}{60}+N(x), & 1\leq x\leq 600\\ \frac{(x-600{)}^{2}}{600}-10+N(x), & 600<x\leq 800 \end{array}\\ \begin{array}{cc} y_{2}=\frac{x}{30}+30\times N(x), & \;\;\;1\leq x\leq 800 \end{array} \end{array}$$

where *x* is a positive integer representing time, and *N*(*x*) is a random fluctuation following the standard normal distribution. The plots of $y_{1}$ and $y_{2}$ are shown in Figure 2. The Spearman and RMI were used to measure the trendability of $y_{1}$ and $y_{2}$. The calculation results are shown in Table 1.

From the results of Spearman in Table 1, we can see that the absolute value of $y_{2}$ was greater than $y_{1}$, indicating that $y_{2}$ had a greater trendability. However, $y_{1}$ is more desirable and more consistent with the actual bearing performance degradation process. For $y_{1}$, the negative trendability of the previous stage and the positive trendability of the later stage offset each other, making the value of Spearman close to 0, which reflects the “misjudgment” of the Spearman coefficient. However, RMI can reduce the impact of previous stage data to a certain extent and prefers the features with good trendability in the later stage. So, RMI is more suitable for degradation feature curve selection. It is worth mentioning that the positive and negative trendability dividing line of ARMI is not necessarily 0, while Spearman is. So, we can use the Spearman coefficient for trendability correction before using ARMI if necessary.

### 2.3. Student’s t-HMM

**Figure 3:**
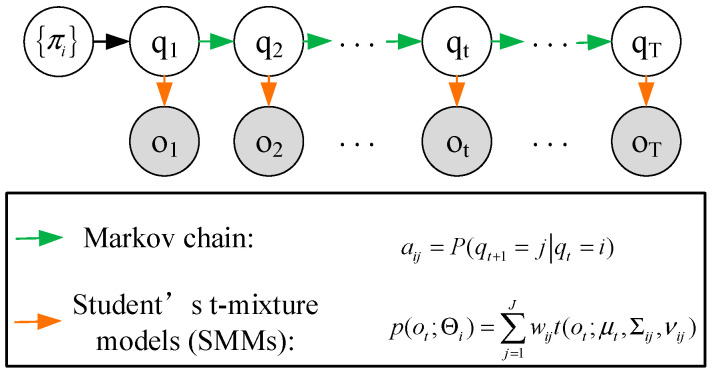
A graphical illustration of Student’s t-HMM.

The hidden Markov model is a dual stochastic model based on time series, which covers two random chains. One is a stochastic process for the observation sequence chain, and the other is a Markov process for the hidden state chain. Based on Bayesian inference, it estimates the hidden state changes from the observation data. Its basic principle is essentially the same as performance degradation evaluation, and the evaluation results are highly interpretable. So, HMMs are widely used in the field of mechanical fault diagnosis and PDA [31,32]. In this study, Student’s t-HMM, which has been proved to be highly tolerant to outliers in real-world applications [27,33], was introduced for bearing PDA based on the selected features. The graphical illustration of Student’s t-HMM is displayed in Figure 3.

The Student’s t-HMM is defined as a finite state-space hidden Markov model whose observation emission distributions of each hidden state are modeled by Student’s t-mixture models (SMMs). Suppose that one Student’s t-HMM has *I* hidden states, and the number of the components of SMMs is *J*. Then, one Student’s t-HMM can be expressed by the following parameters:

- π: the initial state probability distribution. $\pi =\{\pi_{i}\}$, and $\pi_{i}=P(q_{1}=S_{i})$ for 1≤i≤I.
- A: the state transition probability matrix. $A{=\{a}_{ij}\}$, and $a_{ij}=P(q_{t+1}=j|q_{t}=i)$ for 1≤i,j≤I.
- Θ: the observation probability distribution parameter sets based on the Student’s t-HMM. $\Theta =\{\Theta_{i}\}$, and $\Theta_{i}{=\{w}_{ij},\mu_{ij},\Sigma_{ij},\nu_{ij}{\}}_{j=1}^{J}$ for 1≤i≤I. Then, the probability density of the observation $o_{t}$ emitted from the *i*-th hidden state can be calculated as
  $$p(o_{t};\Theta_{i})=\overset{J}{\underset{j=1}{\sum }}w_{ij}t(o_{t};\mu_{t},\sum_{ij},v_{ij})$$

Then, one Student’s t-HMM can be described by π, A, and Θ. For convenience, the notation λ=(π,A,Θ) is used to indicate the complete parameter set of one Student’s t-HMM.

## 3. Bearing PDA Based on SC-RMI and Student’s t-HMM

Raw sensory data exhibit rich degradation information along with many kinds of disturbing information, so it can be quite challenging to obtain effective features with strong trendability to reflect the degradation process in a meaningful way. Moreover, some classical features like RMS lack a stable following trend with degradation process until a few times before failure occurs. Consequently, degradation-sensitive feature extraction and optimal selection from monitoring signals are quite important steps in PDA and have a direct and important impact on the assessment results. According to these, a new PDA framework based on SC-RMI and Student’s t-HMM was proposed. Firstly, spectral clustering was utilized to cluster lifetime feature curves. The features with similar shapes during the whole lifetime were clustered together, and features with different shapes during the whole lifetime were separated from each other. To prevent the loss of effective information, it was necessary to maintain the diversity of feature space. Meanwhile, information redundancy undoubtedly exists among features in the same cluster. Therefore, as the next procedure, optimal features based on RMI from each cluster were selected and put together to construct the final feature set. Feature selection based on these two steps can not only reduce the information redundancy caused by similar feature curves but can also ensure the diversity of different degradation curves in a feature set. So, this feature selection method is called SC-RMI for short. Finally, the selected features were put into Student’s t-HMM for PDA, which covers the training and testing procedures. The main steps are described as follows.

Step 1: feature selection based on SC-RMI. Based on time-frequency domain feature extraction methods, several lifetime feature curves could be obtained from the training lifetime data. Firstly, spectral clustering was utilized to cluster the features with similar shapes and trendabilities during the life cycle. Secondly, in order to fuse the degradation information in different feature clusters and reduce the information redundancy of similar features at the same time, RMI metrics were used to evaluate features, and features with the largest RMI were selected from each cluster. Then, a degradation-sensitive feature set was established with rich degradation information and less redundancy simultaneously. This feature selection method is simply called SC-RMI. 

Step 2: PDA modeling based on Student’s t-HMM. In the application of PDA, a normal Student’s t-HMM is usually constructed based on the degradation-sensitive feature training set from the normal operation state. After the feature selection, the degradation-sensitive feature set was established. Features extracted from data under the normal stage were utilized as the training data for normal Student’s t-HMM modeling. All the algorithms of ordinary HMMs could be applied. Detailed algorithms are provided in reference [34]. Then, a normal Student’s t-HMM could be obtained.

Step 3: performance degradation assessment. The model structure and parameters (λ=(π,A,Θ)) of the normal Student’s t-HMM reflect the multi-state time series statistical law of monitoring data under normal operation state. The degradation process of equipment can be regarded as a deviation from the normal operation state. Then, the testing feature set O is put into the normal Student’s t-HMM (λ) to calculate the likelihood probability output P(O∣λ). The likelihood probability output of the testing data in the trained model is a measure of the membership degree of the state of the testing data to the normal state. The closer the current equipment is to the normal operation state, the greater the likelihood probability of the testing data output in the normal Student’s t-HMM. Therefore, in the framework of equipment PDA based on Student’s t-HMM, the output likelihood probability of the testing data in the normal state model is often recorded as the performance indicator (PI).

**Figure 4:**
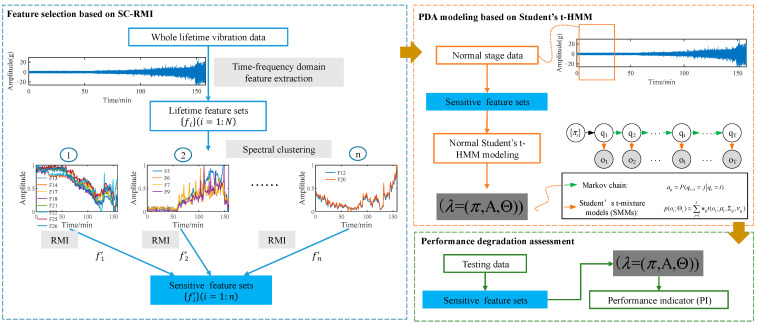
The whole frame of the proposed method.

The whole frame of the proposed method is shown in Figure 4. Sensitive features were selected through SC-RMI as shown in the blue box and used in subsequent steps. The parameters of the trained Student’s HMM calculated in the orange box were passed to the green box for assessment.

## 4. Experimental Verification

To verify the effectiveness of the proposed bearing PDA method, data sets of two bearing-accelerated life tests were utilized

### 4.1. Case Study Ⅰ: An Accelerated Life Test of Rolling Bearings

#### 4.1.1. Experiment Introduction

**Figure 5:**
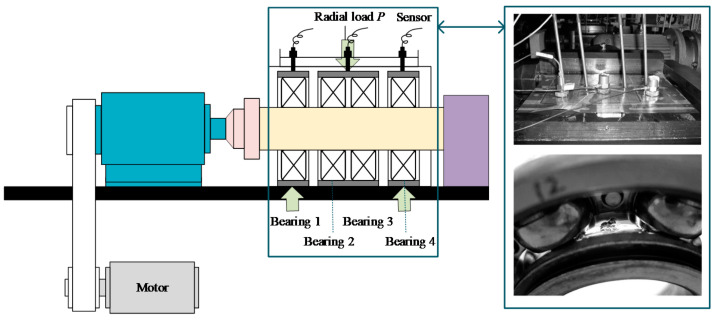
The bearing test-rig and sensors placement illustration.

An accelerated life test of rolling bearings was carried out with the support of the Hangzhou Bearing Test & Research Center (HBRC). The diagram of the test rig is displayed in Figure 5. The platform consists of an AC motor, a transmission system, a lubrication system, a loading system, and a data acquisition system. Four bearings, type 6307, were mounted on the testbed at the same time. The additional load *P* was 12.744 kN, which accelerates the deterioration of the bearing. Three acceleration sensors, type PCB348A, were placed on the shell of the test bearings. Five characteristic frequencies are displayed in Table 2. The sampling frequency was 25.6 kHz, and 20,480 samples (i.e., 0.8 s) were recorded per minute. During the whole test, bearings at Bearing 1 and Bearing 3 locations, as shown in Figure 5, ran to failure first. These two bearings were marked as B1 and B2, separately. So, data sampled from B1 and B2 are utilized in this section. 

#### 4.1.2. Data Description

**Figure 6:**
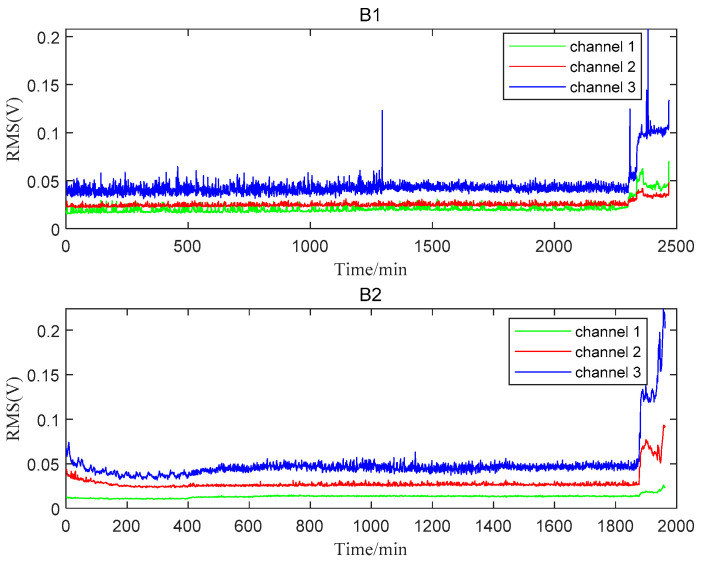
The whole lifetime RMSs of B1 and B2.

The total lifetime of B1 and B2 was 2469 min and 4431 min, respectively. When B1 failed at 2469 min, a new bearing was installed at the position of Bearing 1. At the same time, B2 was found to be in the normal stage. Therefore, only 1962 minutes’ data after replacing B1 were utilized to analyze B2. The RMS plots of B1 and B2 are shown in Figure 6. Channel 3, which was more sensitive to the degradation process for both bearings, was selected for the following analysis. From the perspective of the entire RMS life curve, the two bearings can only be roughly divided into two stages: the normal stage and the failure stage. The B1 bearing entered the failure stage around 2300 min. The early running-in stage could be observed during the first 400 min and the failure stage started around 1850 min for B2. More detailed degradation information could not be caught easily from the RMS plots. 

In practical engineering, time domain and frequency domain statistical features are commonly used in bearing condition monitoring. As a time-frequency analysis method, wavelet packet transform (WPT) is the generalization of the wavelet transform and has been used in several applications of signal processing. The distribution of wavelet packet decomposition node energies relates to the state of bearings. The wavelet packet decomposition node energies and energy entropies are usually extracted as features. It is proved in reference [35] that they have better monotonicity and trendability than traditional features. Envelope spectral entropy and amplitude spectral entropy in information entropy are also used to extract features. Finally, 41 commonly used features were extracted, as shown in Table 3.

#### 4.1.3. Feature Selection Based on SC-RMI

After feature extraction, 41 dimensional original features of B1 could be obtained. Sometimes there will be strong similarity among the extracted features, which means that the fault information they contain is redundant. These features need to be grouped according to their characteristics. Then, 41-dimensional life features extracted from the B1 were clustered using the spectral clustering method. Since the cluster number is an important preset parameter in spectral clustering, it affects the validity of the final selected feature set. In this study, the Fisher discrimination criterion was utilized to determine the optimal number of clusters. The number of clusters was determined when the ratio of the between-class distance and the with-in distance was the largest. In this experiment, the number of clusters was set to 12.

**Figure 7:**
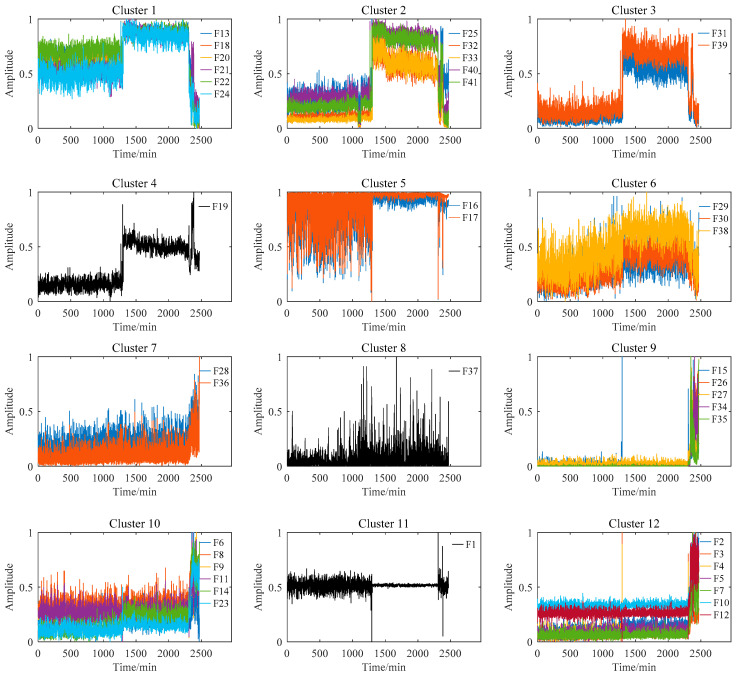
Feature clustering results of bearing B1.

**Figure 8:**
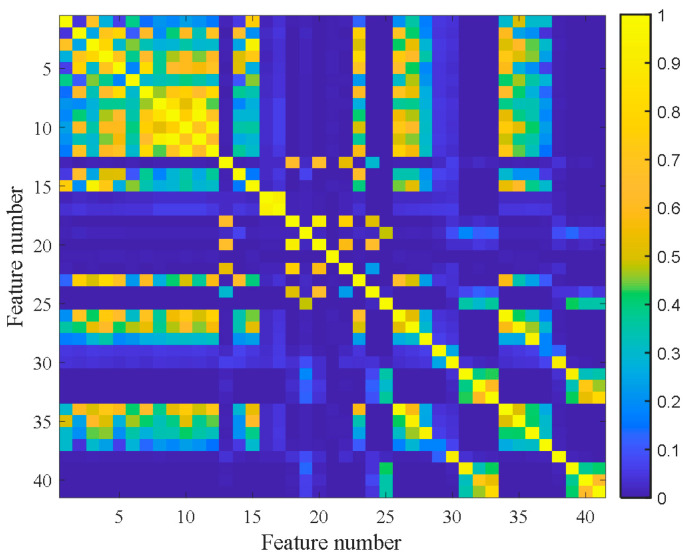
Correlation matrix of bearing B1.

Figure 7 shows the feature clustering results after spectral clustering, and Figure 8 shows their corresponding correlation matrix. It can be seen that they almost overlapped on the same curve, and their degradation information was similar for features in the same cluster. At the same time, it can be seen that some features were sensitive in the severe fault stage. Some features can reflect the early fault information, and some other features cannot even clearly reflect the fault state changes. This was closely related to their feature extraction methods. For example, the features in Cluster 12 were mostly extracted by time domain statistical methods, which are sensitive to the severe fault and insensitive to the early fault. The features in Cluster 5 were extracted based on frequency domain statistical methods, which are only sensitive to the early fault stage. The F37 in Cluster 8 is the node energy of a wavelet packet in a certain frequency band, which fluctuates greatly in the degradation process and has no obvious trend characteristics. None of these features can reflect the complete degradation process alone. Therefore, the features with the largest RMI metrics were selected from each cluster to jointly form the sensitive feature set. This procedure can greatly reduce the information redundancy in the high-dimension feature. At the same time, it retains the diversity of features from different clusters, which is conducive to the construction of PDA health indicators. Table 4 shows the selected features from each cluster and their corresponding RMI values. For the three features with the smallest RMI values that contain less degradation information and more interference, they were not selected. Finally, the top nine sensitive features with high RMI values were selected for further Student’s t-HMM training and assessment.

#### 4.1.4. PDA Based on SC-RMI and Student’s t-HMM

From the RMS diagram of B1, it can be roughly seen that the bearing maintained stable operation in the first 100 min. Therefore, the first 100 min of bearing data were used for normal model training. To better fit the current degradation stage, a model parameter updating strategy was adopted when applying Student’s t-HMM modeling. According to the principle of 3σ criterion, when the distribution of testing data is similar to that of training data, their output PI values in the trained Student’s t-HMM fluctuate in a certain range, which can be calculated based on 3σ criterion. Therefore, it can be considered as the occurrence of a new stage at a certain time if 50 continuous PIs exceed the range of 3σ criterion. However, considering the volatility of data, a 5% fault tolerance rate was set, i.e., 5% points were allowed to fall outside the 3σ criterion. Based on whether the smooth curve had a large continuous decline compared with the previous stable stage, the new stage can be divided into two types: the stable stage and the continuous deterioration stage. Only when the new stage was stable, the first 50 data points of the new stage were used to update the Student’s t-HMM parameters, and the relative likelihood probability curve was calculated since the new stage. Finally, a PI curve reflecting the degradation process of the testing bearing can be obtained.

**Figure 9:**
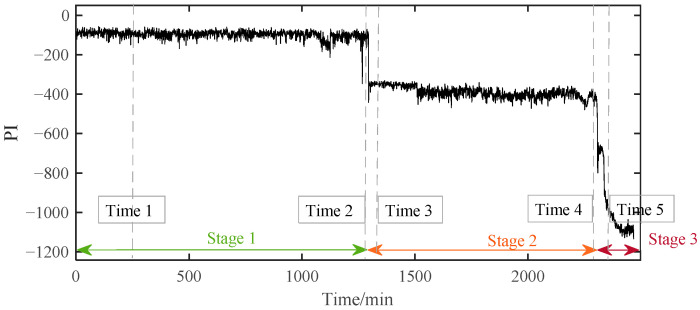
The PI results of B1 based on SC-RMI and Student’s t-HMM.

The PI results of B1 during the whole lifetime can be calculated based on the above method. According to the PI results shown in Figure 9, the whole lifetime of B1 can be divided into three distinct stages. The PI curve kept a stable fluctuation around a certain value during Stage 1 for a long time. So, B1 in this stage was inferred to be under normal running conditions. Then, around 1294 min, the PI value suddenly decreased and then stabilized at a relatively low value. Although the PI value at this time had a significant decrease from −98.6 to about −400, far beyond the normal 3σ range, it kept fluctuating around a relatively stable value in the following period of Stage 2. It can be preliminarily judged that early degradation occurred at the beginning of Stage 2, and the B1 was still running smoothly. Different from the stable changes in the first two stages, the PI value in Stage 3 decreased rapidly until the bearing failed at the end of time, which indicated that the performance of the bearing had changed significantly in a very short time. This is not a possible phenomenon in the stable operation stage and the early failure stage, which means that the bearing had entered the rapid deterioration stage and completely failed at the end of this period.

**Figure 10:**
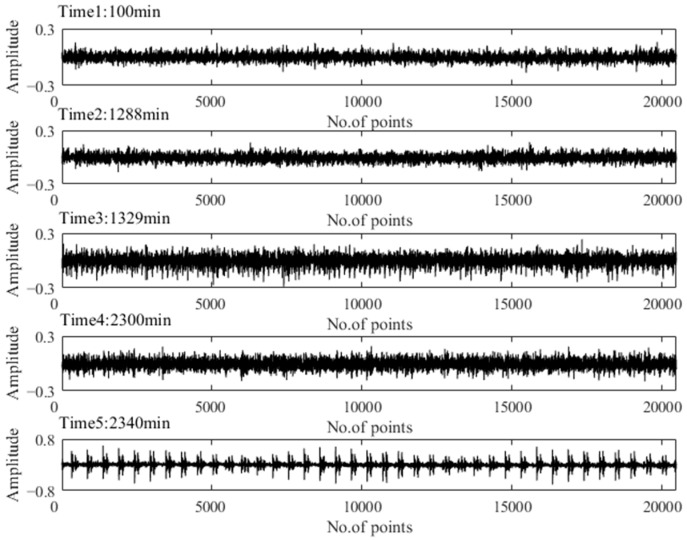
Time plots of five representative moments.

**Figure 11:**
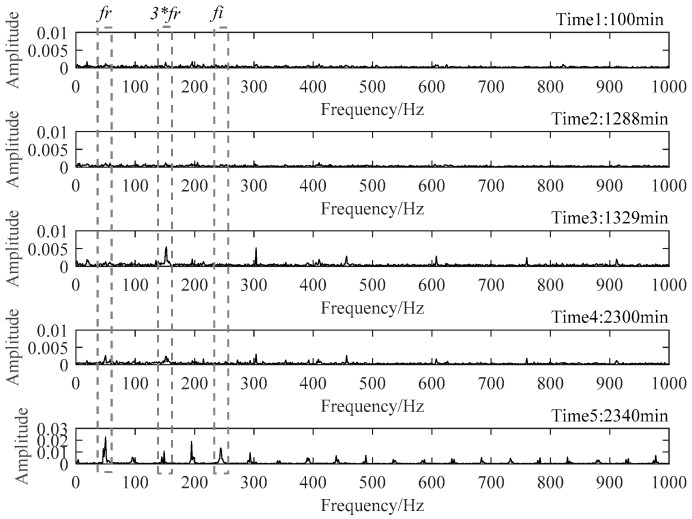
Envelop spectra of five representative moments.

**Figure 12:**
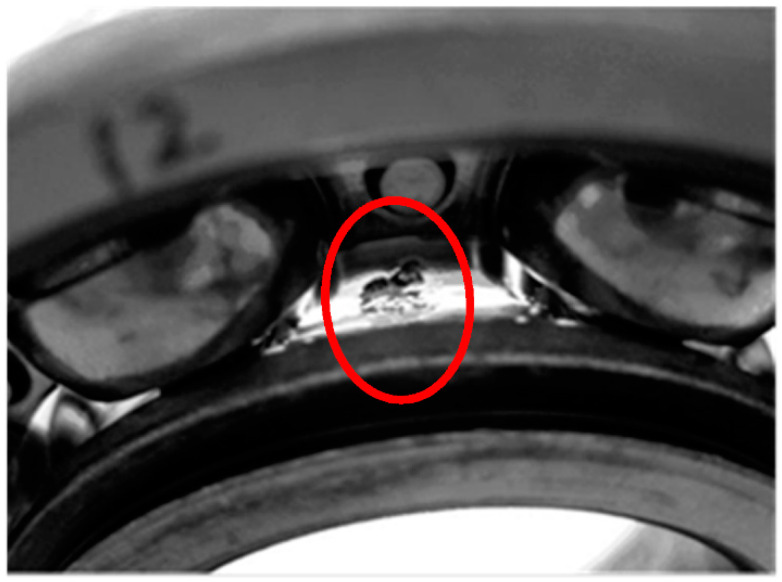
The failure bearing B1.

To verify the above assessment results, five special time points around the stage transition points were chosen for further explanation. Their time plots and envelope spectrum plots are displayed in Figure 10 and Figure 11. At Time 1 and Time 2, no obvious shock signal could be found in the vibration signal, and the amplitudes at the rotating frequency and ball pass inner race frequency (BPIF) could not be observed. At Time 3 and Time 4, the amplitudes of vibration were greater than those at Time 1 and Time 2, and the amplitudes at rotating frequency and its harmonic frequency increased. However, the amplitudes at BPIF were still not obvious. At Time 5, obvious periodic shock could be observed in the time plot, and the amplitudes at the rotating frequency and BPIF were quite obvious. The envelope spectrum covered the characteristics of the bearing inner race fault signal, which means that obvious inner ring fault had occurred in the bearing. B1 did eventually fail for the inner race fault, which was manifested by inner ring surface peeling and serious reduction in surface roughness, as shown in Figure 12, in which the fault position was located in the elliptical red frame. Therefore, PI obtained by the proposed method can effectively evaluate the performance degradation process of B1.

**Figure 13:**
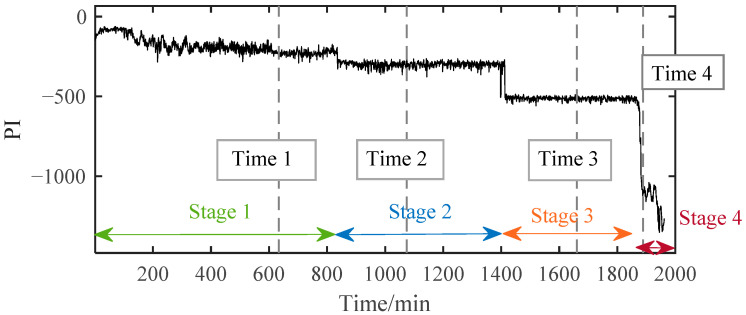
The PI results of B2 based on SC-RMI and Student’s t-HMM.

After assessing the degradation process of B1, these selected degradation-sensitive features were applied to PDA of B2 for further verification. Similarly, the beginning 100 min’ data of B2, which was under the normal condition, were used for normal Student’s t-HMM model training at the beginning. The final assessment results are shown in Figure 13. The entire process of B2 can be divided into four stages. In Stage 1, initially, PI kept at a relatively high level with large fluctuations affected by the replacement of B1. Stage 1 is thought to be a normal stage. Around 836 min, the PI value suddenly decreased, and then it kept stable in a relatively low value, which indicated the occurrence of early degradation recorded as Stage 2. Then, around 1413 min, a similar situation occurred and B2 ran into the next degradation state recorded as Stage 3. The degradation processes in Stage 2 and Stage 3 seemed to be stable, and no obvious fault occurred in B2. Then, around 1871 min, the PI plot decreased rapidly in a very short time until B2 ran to failure. As in the case of B1, the PI value of B2 in Stage 4 decreased rapidly until the failure was recognized as the rapid deterioration stage.

**Figure 14:**
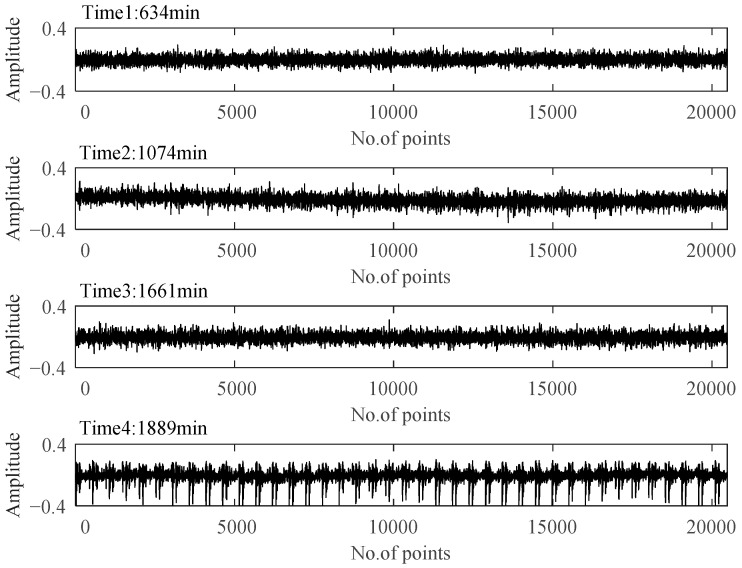
Time plots of four representative moments.

**Figure 15:**
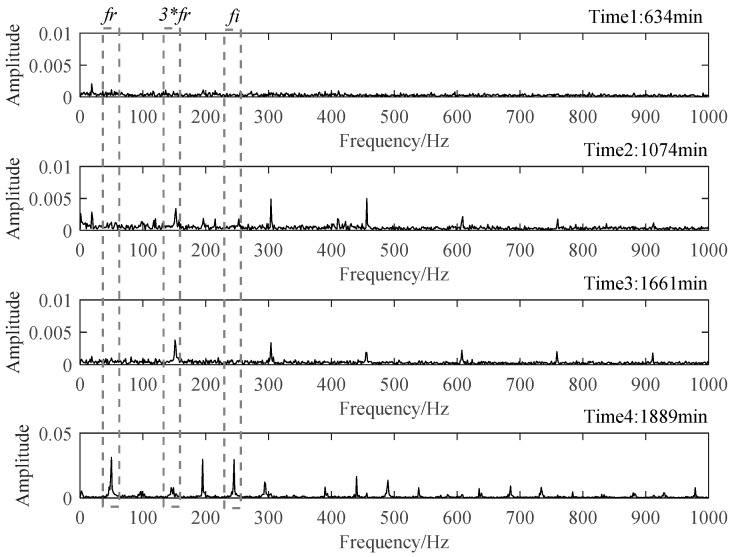
Envelop spectra of four representative moments.

**Figure 16:**
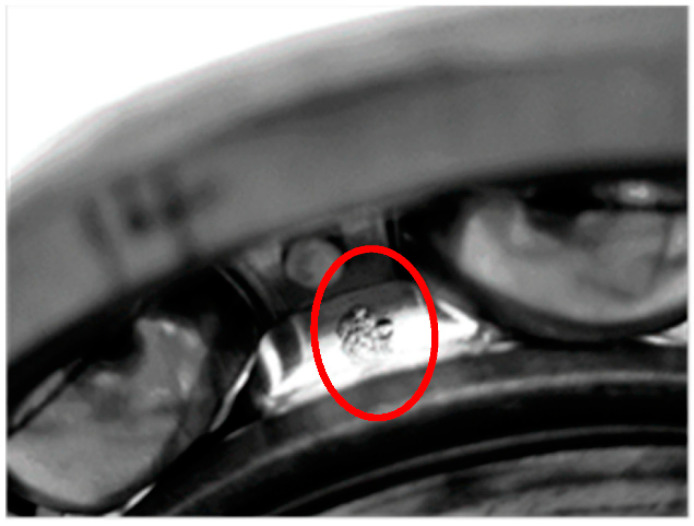
The failure bearing B2.

The time plots and envelope spectra of four representative moments are shown in Figure 14 and Figure 15. At Time 1, any fault shock characteristic could not be observed from both the time plot and the envelop spectrum. For signals at Time 2 and Time 3, they were quite similar to each other in both time plots and envelop spectra. The amplitudes at rotating frequency and its harmonic frequency were quite outstanding compared with Time 1, and the amplitudes at BPIF were still unclear. However, the amplitude distributions in the frequency domain were different between them. So, different degradation stages could be separated for Stage 2 and Stage 3. At Time 4, obvious periodic shock could be observed in the time plot, and the amplitudes at the rotating frequency and BPIF were quite obvious, which matched the characteristics of the bearing inner race fault signal. B2 did eventually fail for the inner race fault, as shown in Figure 16, which was the failure mode similar to bearing B1. Therefore, the selected features were proved to be effective for the performance degradation process of B2 too.

#### 4.1.5. Comparisons with Other Methods

To further verify the effectiveness of our proposed PDA method, it was compared with the other two methods. One was to use full features for assessment, and the other was to use RMI directly to select features for assessment without using spectral clustering. Other feature extractions and parameter selection procedures were kept the same.

**Figure 17:**
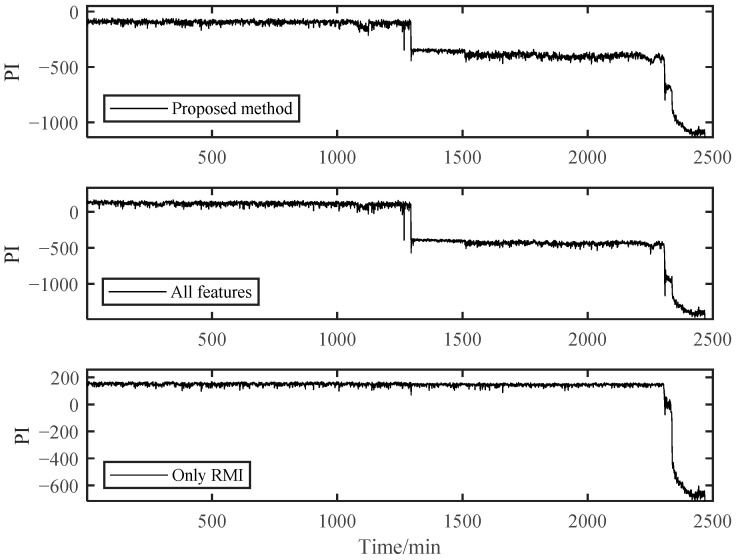
Comparison of three different methods of B1.

**Figure 18:**
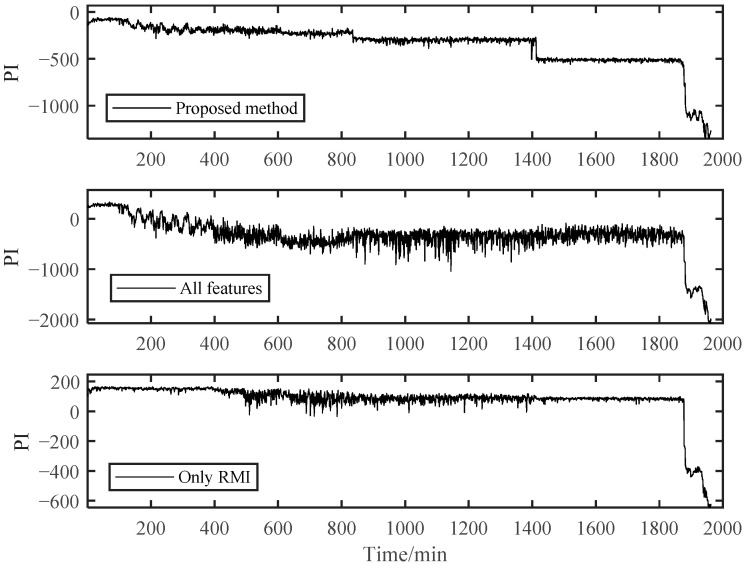
Comparison of three different methods of B2.

As shown in Figure 17, the PI results of B1 obtained using the RMI-only method for feature selection could not distinguish the early degradation stage. However, there was a smooth curve in the severe degradation stage, which shows the applicability of RMI in PDA. Both the fully-featured training model and our proposed method could distinguish different stages, but the PI value of the fully-featured method essentially had greater fluctuations, although it looks the opposite in Figure 17. For the B2 bearing shown in Figure 18, it may experience a running-in period in the beginning because B2 data were collected after replacing B1. This shows a relatively violent shock compared to the normal operation stage in B1. However, it was difficult for us to directly distinguish it from the normal stable operation phase due to the unobvious running-in period. Therefore, the degradation curves obtained by the other two methods were difficult to distinguish between the normal operation stage and the early degradation stage, let alone to observe the weak failure changes that may occur in the early degradation stage. However, our proposed method can sensitively capture the change in the bearing degradation stage, which illustrates the robustness of our method.

Different metrics of PIs obtained by these three methods are provided in Table 5, among which the calculation formulas for trendability and robustness can be found in [1]. The trendability in Table 5 was obtained by using the Spearman coefficient. For the B1 bearing, the proposed method performed better in RMI and trendability metrics. The only RMI method showed better on robustness metrics. RMI was more sensitive to the data in the later stage, so it tended to have better robustness. However, this may lead to its failure in early fault stage division. For the B2 bearing, these three metrics values of the proposed method were the largest. In conclusion, the proposed method achieved better results than the other two methods in these three metrics.

### 4.2. Case Study Ⅱ: The Public XJTU-SY Bearing Data Set

#### 4.2.1. Experiment Introduction and Data Description

**Figure 19:**
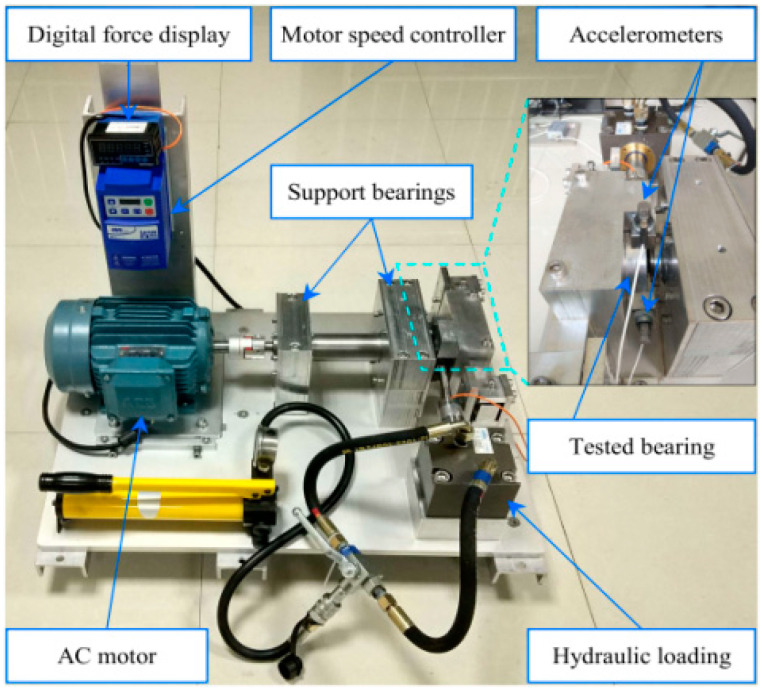
Bearing testbed.

**Figure 20:**
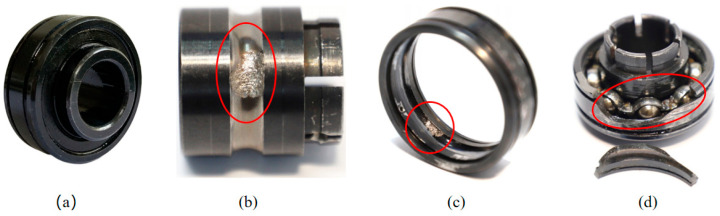
Photos of tested bearings: (**a**) normal bearing; (**b**) inner race wear; (**c**) outer race wear; (**d**) outer race fracture.

This article adopted the XJTU-SY data set of the accelerated life test for rolling bearings [37]. The bearing testbed is shown in Figure 19. This platform can conduct accelerated degradation tests of bearings to provide real experimental data that characterize the degradation of bearings during the whole operating life. Fifteen rolling bearings of LDK UER204 were tested under three different working conditions. The material of LDK UER204 is GCr15, which has a high and uniform hardness after quenching and tempering, a high wear resistance, contact fatigue resistance, and good hot workability. It is often used in bearing production. The detailed parameters are given in Table 6. The failure of the bearings in the test was caused by different types of faults like inner race wear, outer race wear, outer race fracture, etc. Figure 20 displays the photos of normal and typical failure bearings. To acquire the run-to-failure data of the tested bearings, as shown in Figure 18, two PCB 352C33 accelerometers were placed on the housing of the tested bearings and positioned at 90° to each other, i.e., one was placed on the vertical axis and the other one was placed on the horizontal axis. The sampling frequency was 25.6 kHz, and 32,768 samples (i.e., 1.28 s) were recorded every 1 min. Besides, the accelerated degradation tests of bearings were stopped when the amplitude of the vibration signal was higher than 20 g. Correspondingly, the time when the amplitude of the vibration signal exceeded 20 g was considered as the failure time of the tested bearing.

**Figure 21:**
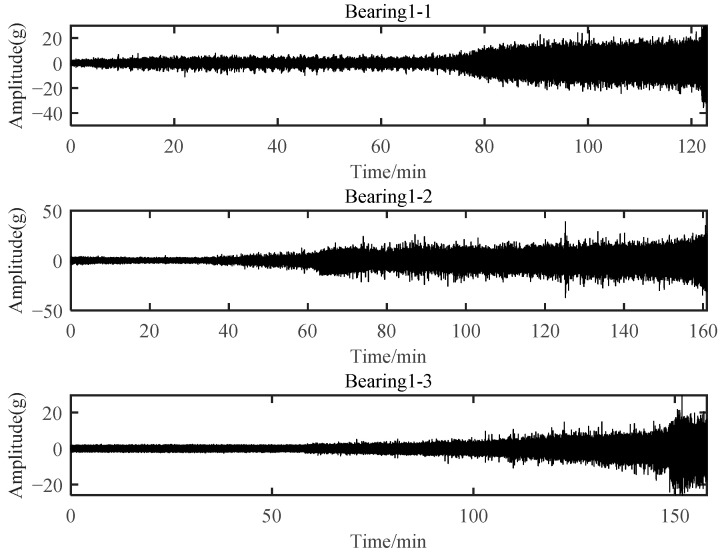
Horizontal vibration signal of three bearings.

Bearings with an outer race fault during failure under running condition 1 were selected for verification, which included Bearing 1-1, Bearing 1-2, and Bearing 1-3. Specifically, Bearing 1-1 was used to select degradation-sensitive features based on SC-RMI. All these three bearings were further used to verify the effectiveness of the proposed PDA method. Figure 21 shows the horizontal vibration signal of these three bearings. 

#### 4.2.2. Feature Selection Based on SC-RMI

**Figure 22:**
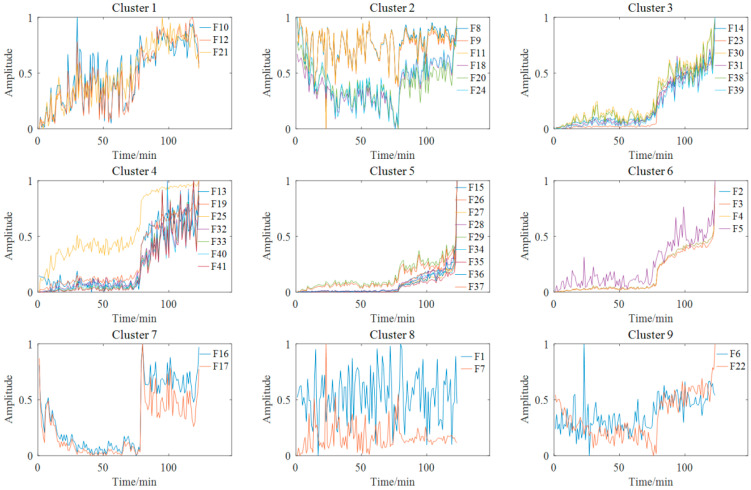
Feature clustering results of Bearing 1-1.

**Figure 23:**
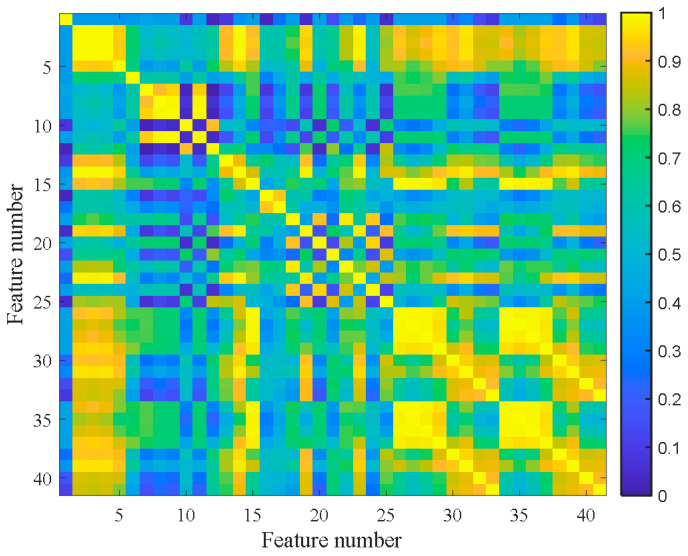
Correlation matrix of Bearing 1-1.

The 41-dimensional original features were extracted according to the feature extraction used in case study Ⅰ. The number of clusters was set to nine according to the Fisher discrimination criterion. Figure 22 shows the feature clustering results after spectral clustering, and Figure 23 shows the corresponding correlation matrix of Bearing 1-1. It can be seen that the curves of features in the same cluster are similar or even overlapped, which indicates the effectiveness of spectral clustering in feature clustering. Some features were suitable for PDA, while others were not. The poor metrics may also contain key degraded information. For example, there was an obvious stage division in Cluster 7, which is useful for PDA. We can tolerate these features if it does not affect the final trendability of PI. Moreover, too many similar features will cause the PDA results to be overly affected by a single type of feature, thus losing the diversity of features. So, SC-RMI was utilized for feature selection in PDA. Table 7 shows the selected features from each cluster and their corresponding RMI values. It can be seen that the RMI value of feature F1 from Cluster 8 was far less than the other values. At the same time, F1 contained little degradation stage information and fluctuated greatly compared with other features. So, F1 was removed. Finally, the top eight sensitive features with high RMI values were selected for Student’s t-HMM training and assessment. 

**Figure 24:**
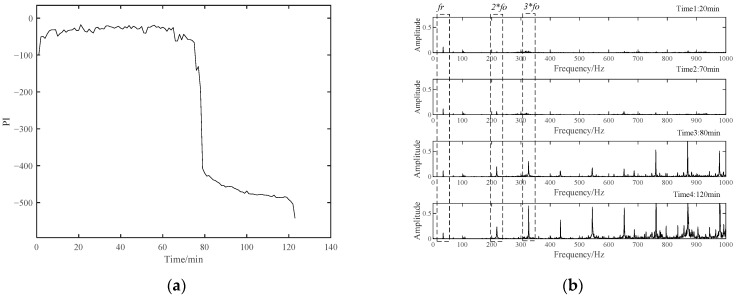
(**a**) The PI results of Bearing1-1; (**b**) envelop spectra of four representative moments.

The data of the first 50 min of Bearing 1-1 were used for Student’s t-HMM model training, and the PI results during the whole lifetime are displayed in Figure 24a. In about 75 min, the bearing began to enter a rapid deterioration stage and maintained a stable degradation process until failure. The envelope spectra of four representative moments are shown in Figure 24b. The spectra of Time 1 and Time 2 were similar, and the envelope spectrum mainly included the rotation frequency component of the bearing. At this time, amplitudes at the outer race fault frequency and its multiple frequency were not obvious. From Time 3 to Time 4, the multiple frequency components of the bearing outer race fault became more and more obvious. This was consistent with the PI results of Bearing1-1.

#### 4.2.3. Comparisons with Other Methods

**Figure 25:**
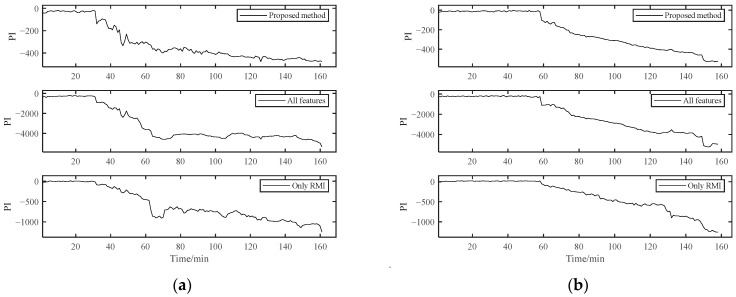
Comparisons with different methods: (**a**) Bearing 1-2; (**b**) Bearing 1-3.

The selected features of Bearing 1-1 were used for the PDA of Bearing 1-2 and Bearing 1-3 for further verification. At the same time, PDA based on the method with all features and the method with feature selection only by RMI were carried out for comparison. Except for the different methods of feature selection, other parameters and techniques were kept the same. PI curves calculated by these three methods are plotted in Figure 25. For Bearing 1-2, the PI curve obtained by the proposed method achieved better performance. The PI curves obtained by the other two methods contained more fluctuations, especially during 60 min to 80 min. For Bearing 1-3, the results of the proposed method were still better than the other two methods. Unexpected excessive mutations existed in PI curves since around 120 min. Three different metrics were calculated for PIs of these three methods, which are shown in Table 8. The proposed method achieved better results than the other two methods in three metrics. In addition, the method with all features had a larger amount of calculation, and the method with feature selection only by RMI could not ensure the diversity of feature sets. With the help of SC-RMI, the proposed method not only made full use of the diversity of features but also reduced the interference of redundant information. Therefore, the bearing PDA based on SC-RMI and Student’s t-HMM can achieve better performance. 

## 5. Conclusions

An effective bearing performance degradation assessment method based on SC-RMI and Student’s t-HMM was proposed in this study. Spectral clustering was firstly used to cluster high-dimensional features, which showed outstanding performance in similar lifetime curve clustering. Rank mutual information, which is more suitable for trendability estimation of long time series, was utilized to select optimal features from each cluster. Based on the cooperation of these two techniques, features with good trendability for the bearing degradation process and less redundant information can be selected. Finally, Student’s t-HMM was utilized for the degradation process modeling and assessment, which showed strong robustness in PDA. The verification by accelerated bearing degradation experiment and the public XJTU-SY dataset showed the superiority of our proposed method with the advantages of sensitivity to degradation, good trendability, and strong robustness. In particular, the performance indicator obtained based on the proposed method had a good trendability in the fault deterioration stage, which is very valuable for later remaining useful life prediction. In addition, the proposed method of this study can contribute to the development of equipment intelligent maintenance in the era of big data and intelligent manufacturing. 

## Figures and Tables

**Table 1:** The trendability evaluated by Spearman coefficient and ARMI.

Data	Spearman Coefficient	ARMI
$y_{1}$	−0.0955	0.2689
$y_{2}$	0.2262	0.1706

**Table 2:** Characteristic frequencies of rolling element bearing 6307.

Type	f_r_	f_bp_	f_ip_	f_op_	f_c_
6307	50 Hz	102 Hz	246 Hz	153 Hz	19 Hz

**Table 3:** 41-Dimension features.

Feature Type	Detailed Feature	
Time-domain feature	F1: average value	F7: Kurtosis factor
	F2: standard deviation	F8: Skewness factor
	F3: square root amplitude	F9: Crest factor
	F4: root mean square	F10: Margin factor
	F5: peak	F11: Impulse factor
	F6: skewness	
Entropy feature	F12: envelope spectral entropy	F13: amplitude spectral entropy
Frequency-domain feature	F14–F25: full details are given in reference [36]	
Time-frequency feature	F26–F33: wavelet packet energy	
	F34–F41: wavelet packet energy entropy	

**Table 4:** RMI Values of the selected features.

Feature Number	14	2	26	28	19	30
value	0.60	0.54	0.39	0.35	0.28	0.23
Feature number	31	25	22	37	17	1
value	0.16	0.13	0.12	0.07	−0.01	−0.10

**Table 5:** Three different metrics of PIs by different methods.

Bearing Number	Different Methods	RMI	Trendability	Robustness
B1	Proposed method	0.78	0.87	0.91
	All features	0.77	0.87	0.87
	Only RMI	0.47	0.41	0.94
B2	Proposed method	0.81	0.95	0.93
	All features	0.40	0.48	0.79
	Only RMI	0.53	0.71	0.88

**Table 6:** Parameters of the tested bearings.

Parameter	Value	Parameter	Value
Outer race diameter	39.80 mm	Inner race diameter	29.30 mm
Bearing mean diameter	34.55 mm	Ball diameter	7.92 mm
Number of balls	8	Contact angle	0°
Load rating (static)	0.88	Load rating (dynamic)	12.82 kN

**Table 7:** RMI of Bearing 1-1 optimal features.

Feature Number	23	2	15	19	12	22	18	16	1
Value	0.88	0.87	0.87	0.85	0.65	0.59	0.5	0.38	0.1

**Table 8:** Three different metrics of PIs by different methods.

Bearing Number	Different Methods	RMI	Trendability	Robustness
Bearing 1-2	Proposed method	0.90	0.98	0.92
	All features	0.70	0.84	0.92
	Only RMI	0.83	0.95	0.89
Bearing 1-3	Proposed method	0.91	0.94	0.91
	All features	0.86	0.95	0.88
	Only RMI	0.89	0.93	0.89

## Data Availability

Not applicable.
